# Paget’s Disease and Secondary Hyperparathyroidism: Is Healing Possible?

**DOI:** 10.3389/fcell.2020.00399

**Published:** 2020-05-29

**Authors:** Vincenzo Antonio Panuccio, Rocco Tripepi

**Affiliations:** ^1^Nephrology, Dialysis and Transplantation Unit G.O.M. “Bianchi Melacrino Morelli”, Reggio Calabria, Italy; ^2^Institute of Clinical Physiology (IFC-CNR), Research Unit of Reggio Calabria, Reggio Calabria, Italy

**Keywords:** Paget bone disease, hyperparathyroidism, vitamin D, alkaline phosphatase, CKD

## Abstract

Paget bone disease (PDB) is often asymptomatic and incidentally diagnosed. It is a cause of osteoporosis and bone fragility and exposes patients to a high incidence of bone fractures. In Europe the prevalence varies according to the geographical area of origin, and increases with age. In patients with chronic renal disease, the prevalence is unknown and only few cases with PDB have been reported. We present a challenging case in an elderly patient with chronic kidney disease on peritoneal dialysis treatment. Our patients presented extremely high levels of alkaline phosphatase, suggesting a Paget bone disease. Secondary hyperparathyroidism was confirmed by the bone histological examination. The surprising biochemical and clinical response to active vitamin D confirms the well-known role on hyperparathyroidism and may indicate an additional role in the pathogenesis of Paget’s disease.

## Introduction

Paget bone disease (PDB) is a cause of osteoporosis and bone fragility and exposes patients to a high incidence of bone fractures (Dove, 1980; Melton et al., 2000). It is characterized by an imbalance between resorption and deposition of new bone tissue (Iaquinta et al., 2019) and by abnormal osteoclasts (OCL) that secrete high IL-6 levels and induce exuberant bone formation. The altered remodeling results in formation of unstructured, fibroblastic and mechanically fragile bone tissue. Its etiopathogenesis could be linked, at least in part, to infectious factors (Kurihara et al., 2011). A possible relationship between a mutation on chromosome 5 and Paget’s disease of bone has recently been described (Michou et al., 2006). In Europe, the prevalence varies from 0.1 to 2% of the adult population, depending upon the geographical area of origin, and increases with age (Poór et al., 2006).

In a high proportion of cases, PDB is discovered incidentally on biochemical testing or radiography. It is possible to detect an isolated increase in alkaline phosphatase (ALP), proportional to the extension of bone disease. Though generally asymptomatic, pain at rest is the most common symptom. A very small percentage of patients may experience a dreadful complication of sarcomatous degeneration.

Paget bone disease can occur in two forms: monostotic if a single bone in involved, or polyostotic if several bone areas are affected. Any bone in the skeleton can be involved, the most frequent being the pelvis, spine, sacrum, skull, femora, and tibiae. In patients with chronic renal disease, the prevalence is unknown. Secondary hyperparathyroidism is a frequent condition in dialysis patients and can easily mask PDB diagnosis. Furthermore, in dialysis patients, the existing association between bone fractures and cardiovascular comorbidities (Fusaro et al., 2013) is well known as is the predictive role of high iPTH levels and pro-inflammatory cytokines, such us TNF-α, in new bone fractures (Panuccio et al., 2012).

We present a challenging case of PBD in an elderly patient with chronic kidney disease (CKD) on peritoneal dialysis.

## Case Report

In 1989, a 60-year-old man with stage 4 CKD with a history of hypertension was referred to our Nephrology and Transplant unit for generalized bone pain and functional limitation. The patient had CKD stage 3b for 9 years until he was admitted in a Pulmonary Unit for tuberculosis disease treated with specific therapy. He also had uncomplicated diabetes mellitus on insulin therapy. Radiographs showed reduced kidney measures. Proteinuria was less than 1 gr/24 h. Koch bacillus in the urine was not detected. Differential diagnosis of the nephropathy was clinical and postulated as secondary to vascular damage, such as nephroangiosclerosis. Patient presented with hypocalcemia (7.6 mg/dl), normal phosphorus levels (average 3.6 mg/dl) and high iPTH (700 pg/ml) and Alkaline Phosphate (AP) (1600 UI/L) levels. He received a calcium-based phosphate binder and active vitamin D supplements thus reaching normalization of serum calcium levels but with a significant increase in phosphate and AP levels (the latter from 1600 to 6000 UI/L).

**FIGURE 1:**
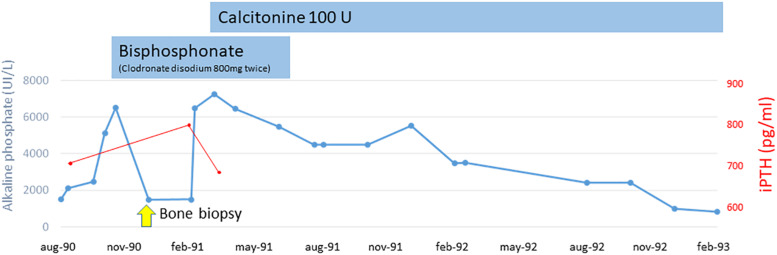
Alkaline phosphatase and intact parathyroid hormone trend during pre-dialysis period.

Bone pain become disabling and radiographs showed deformity of long bones and typical “cotton wool” appearance of the skull. Before excluding a possible neoplastic origin of high AP levels and, on the basis of the clinical and laboratory data, we suspected PDB and decided to treat our patient empirically with bisphosphonate (clodronate disodium 800 mg twice). We observed a rapid reduction of AP levels (to 1800 UI/L). A bone biopsy was suggestive of PDB with superimposed lesions typical of hyperparathiroidism. Alluminum staining was negative. After 3 months, we observed a new increase of AP levels (7000 UI/L) so clodronate was interrupted and nasal calcitonin was prescribed for 6 months resulting in a reduction of AP levels (1000 UI/L). AP and iPTH trends during the pre-dialysis period are shown in Figure 1.

In May, 1993, the patient started continuous ambulatory peritoneal dialysis (CAPD). Three months after suspension of calcitonin, the AP level returned near 5000 UI/L and bone pain remained severe. At this point, treatment was focused on the component of secondary hyperparathyroidism, and oral vitamin D boluses (calcitriol 1 mcg once day) were prescribed.

**FIGURE 2:**
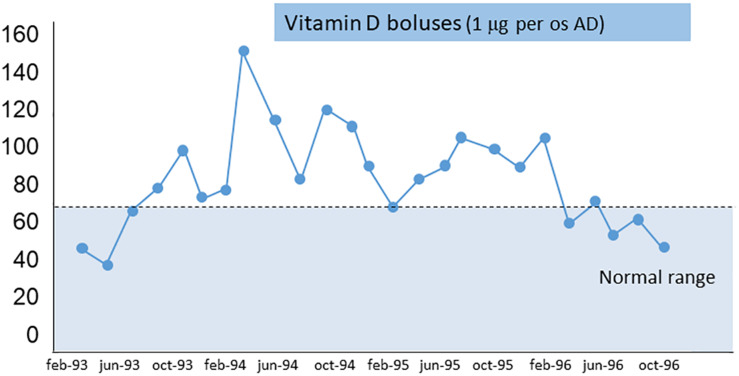
Calcium-phosphate product (mg^2^/dL^2^) trend during vitamin D treatment.

**FIGURE 3:**
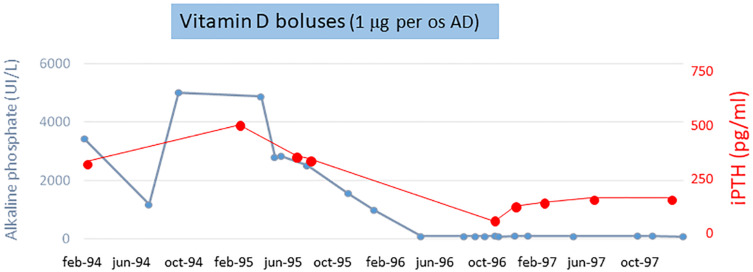
Alkaline phosphatase and intact parathyroid hormone trend during dialysis period.

The AP and iPTH levels progressively decreased and reached normal range (AP about 80 UI/L and iPTH <250 pg/ml) at 6 months. Figure 2 shows the calcium-phosphate product (CaxP) trend during the same period, after starting vitamin D treatment CaxP progressively decrease to became in the normal range. Treatment was stopped with a “watch and wait” approach; to our extreme surprise indicators of bone metabolism remained stable for more than 1 year (AP and iPTH trends during dialysis period are shown in Figure 3). To evaluate histological changes in bone turnover, we performed a second bone biopsy at the end of 1997. The bone framework was generally unchanged from the previous one, but with a decrease in severity of bone lesions.

## Discussion

Severe bone pain associated with osteoarticular functional limitation in patients with chronic kidney disease is not common. The extremely high levels of alkaline phosphatase in our patient suggested a Paget bone disease. The peculiarity of our clinical case is that at the same time there was a serious secondary hyperparathyroidism, as confirmed by the bone histological examination. The surprising biochemical and clinical response to active vitamin D (Guillard Cumming et al., 1985; Beresford et al., 1986) may confirm his involvement importance in the pathogenesis of pagetic disease and naturally on the component of hyperparathyroidism. Active vitamin D represents a useful and manageable alternative to the bisphosphonate on the treatment of this patients on dialysis to treat these double disease that induces bone fragility and significantly increases the risk of bone fractures.

Serum ALP is recommended as a first line biochemical screening test, in combination with liver function tests, in screening for the presence of metabolically active PDB.

For the management of PDB, it is important to perform radionuclide bone scans, in addition to targeted radiographs, recommended as a means of fully and accurately defining the extent of metabolically active disease. Treatment in asymptomatic patients is controversial. In the randomized PRISM study, there was no benefit in terms of reducing the incidence of fractures, quality of life and bone pain in attempting to normalize ALP in asymptomatic patients (Langston et al., 2010). Anti-resorptive therapy consists of bisphosphonates for most patients with active PDB who are at risk for future complications. For those intolerant to bisphosphonates, subcutaneous calcitonin can be used for a limited period due to its associated risk of malignancy with long-term use (Ralston et al., 2019). Bisphosphonate treatment may be effective in preventing or slowing the progress of hearing loss and osteoarthritis in joints adjacent to PDB and may reverse paraplegia associated with spinal Paget disease. The adverse events that have been reported with bisphosphonate treatment relevant to the treatment of PDB are rare and include a typical femoral fractures, uveitis, osteonecrosis of the jaw, hypocalcemia, and impaired renal function. Case reports of the use of denosumab 60 mg by subcutaneous injection every 6 months in PDB patients in which bisphosphonates were poorly tolerated or contraindicated, a decrease in total ALP concentrations and an improvement of bone pain was observed (Ralston et al., 2019). There are limited reports on the treatment of patients with CKD and PBD. However, the 2017 KDIGO guideline suggests aggressive control of serum parathyroid hormone, calcium, phosphorus, and vitamin D level for the CKD-MBD patient (Isakova et al., 2017). In this field, calcium mimetics are useful to reach the targets for bone mineral metabolism parameters.

It is important to individualize therapy and to try to prevent falls with regular exercise to maintain joint mobility and bone strength. Patient management with PDB and CKD is a challenge because they are synergistic in affecting bone quality and increasing the risk of fractures.

## Data Availability Statement

The datasets generated for this study are available on request to the corresponding authors.

## Ethics Statement

Written informed consent was obtained from the individual for the publication of any potentially identifiable images or data included in this article.

## Author Contributions

VP conceived the study. VP and RT collected information, drafted the manuscript and approved the final manuscript.

## Conflict of Interest

The authors declare that the research was conducted in the absence of any commercial or financial relationships that could be construed as a potential conflict of interest.

## References

[B1] Beresford J. N. Gallagher J. A. Russell R. G. (1986). 1,25-Dihydroxyvitamin D3 and human bone-derived cells in vitro: effects on alkaline phosphatase, type I collagen and proliferation. *Endocrinology* 119 1776–1785. 10.1210/endo-119-4-1776 3489608

[B2] Dove J. (1980). Complete fractures of the femur in paget’s disease of bone. *J. Bone Joint Surg.* 62 12–17. 10.1302/0301-620x.62b1.7351428 7351428

[B3] Fusaro M. Tripepi G. Noale M. Vajente N. Plebani M. Zaninotto M. (2013). High prevalence of vertebral fractures assessed by quantitative morphometry in hemodialysis patients, strongly associated with vascular calcifications. *Calcif Tissue Int.* 93 39–47. 10.1007/s00223-013-9722-x 23494409

[B4] Guillard Cumming D. F. Beard D. J. Douglas D. L. Johnson S. K. Awson Matthew P. J. Russell R. G. G. (1985). Abnormal vitamin d metabolism in Paget’s disease of bone. *Clin. Endocrinol.* 22 559–566. 10.1111/j.1365-2265.1985.tb00157.x 3872746

[B5] Iaquinta M. R. Mazzoni E. Bononi I. Rotondo J. C. Mazziotta C. Montesi M. (2019). Adult stem cells for bone regeneration and repair. *Front. Cell Dev. Biol.* 12:268. 10.3389/fcell.2019.00268 31799249 PMC6863062

[B6] Isakova T. NicKolas T. L. Denburg M. Yarlagadda S. Weiner D. E. Gutierrez O. M. (2017). KDIGO clinical practice guideline uptdate for the diagnosis, evaluation, prevention, and Treatment of Chronic Kidney Disease-Mineral and Bone Disorder (CKD-MBD). *AJKD* 70 737–751. 10.1053/j.ajkd.2017.07.019 28941764

[B7] Kurihara N. Hiruma Y. Yamana K. Michou L. Morissette J. Galson D. L. (2011). Contributions of the measles virus nucleocapsid gene and the SQSTM1/p62P392L mutation to Paget’s disease. *Cell Metab.* 13 23–34. 10.1016/j.cmet.2010.12.002 21195346 PMC3025409

[B8] Langston A. L. Campbell M. K. Raser W. D. MacLennan G. S. Selby R. Pelston S. H. (2010). Randomized trial of intensive bisphosphonate treatment versus symptomatic management in Paget’s disease of bone. *J. Bone Miner. Res.* 25 20–31. 10.1359/jbmr.090709 19580457

[B9] Melton L. J. Tiegs R. D. Atkinson E. J. O’Fallon W. M. (2000). Fracture risk among patients with Paget’s disease: a population-based cohort study. *J. Bone Miner. Res.* 15 2123–2128. 11092393 10.1359/jbmr.2000.15.11.2123

[B10] Michou L. Collet C. Laplanche J. L. Orcel P. Cornélis F. (2006). Genetics of Paget’s disease of bone. *Joint Bone Spine* 73 243–248. 16574459 10.1016/j.jbspin.2005.05.009

[B11] Panuccio V. Enia G. Tripepi R. Aliotta R. Mallamaci F. Tripepi G. (2012). Pro-inflammatory cytokines and bone fractures in CKD patients. An exploratory single centre study. *BMC Nephrol.* 9:134. 10.1186/1471-2369-13-134 23043229 PMC3472278

[B12] Poór G. Donáth J. Fornet B. Cooper C. (2006). Epidemiology of Paget’s disease in Europe: the prevalence is decreasing. *J. Bone Miner. Res.* 21 1545–1549. 10.1359/jbmr.060704 16995808

[B13] Ralston S. H. Corral-Gudino L. Cooper C. Francis R. M. Fraser W. D. Gennari L. (2019). Diagnosis and management of Paget’s disease of bone in adults: a clinical guideline. *J. Bone Miner. Res.* 34 579–604. 30803025 10.1002/jbmr.3657 PMC6522384

